# Risk assessment validation in patients with pulmonary arterial hypertension: Data from a Southern Brazil registry (RESPHIRAR study)

**DOI:** 10.1002/pul2.12193

**Published:** 2023-01-01

**Authors:** Fernanda Brum Spilimbergo, Roger Pirath Rodrigues, Marcelo Credidio Dias‐Pinto, Daniela Cavalet Blanco, Gláucia Maria Barbieri, Marina Andrade‐Lima, Ariovaldo Leal Fagundes, Marcelo Basso Gazzana, Gabriela Roncato, Marcelo Martins Mello, Guilherme Watte, Taís Silveira Assmann, Cássia Ferreira Braz Caurio, Rogerio Souza, Gisela Martina Bohns Meyer

**Affiliations:** ^1^ Centro de Hipertensão Pulmonar, Complexo Hospitalar Santa Casa de Misericórdia de Porto Alegre Porto Alegre Brazil; ^2^ University Hospital of the Federal University of Santa Catarina (UFSC) Florianópolis Brazil; ^3^ Hospital Pequeno Príncipe Curitiba Brazil; ^4^ School of Medicine, Pontifícia Universidade Católica do Rio Grande do Sul (PUCRS) Porto Alegre Brazil; ^5^ Hospital de Clínicas do Paraná Curitiba Brazil; ^6^ Hospital Dia do Pulmão Blumenau Santa Catarina Brasil; ^7^ Pulmonology Department of the Santa Maria University Hospital Federal University of Santa Maria Santa Maria Brazil; ^8^ Pulmonology Unit—Hospital de Clínicas de Porto Alegre (HCPA) Porto Alegre Brazil; ^9^ Divisão de Pneumologia, Instituto do Coração, Hospital das Clínicas HCFMUSP, Faculdade de Medicina da Universidade de São Paulo São Paulo Brazil

**Keywords:** pulmonary arterial hypertension, risk assessment, Southern Brazil registry

## Abstract

Pulmonary arterial hypertension (PAH) is a severe and progressive disease characterized by increased pulmonary vascular resistance, ultimately leading to right heart failure and death. Registries are a valuable tool in the research of rare conditions such as PAH. Moreover, the risk assessment strategy has been validated in European and North American registries and has been reported to provide an accurate prediction of mortality and the clinical advantage of reaching low‐risk status. However, there is no available data from Brazil. Thus, the aim of the present study was to describe the characteristics of a sample of PAH from Southern Brazil and to retrospectively validate the risk assessment at our population. The RESPHIRAR is a retrospective and multicentric registry on pulmonary hypertension. With a join collaboration from nine centers in Southern Brazil, demographics, clinical presentation, and hemodynamics data of PAH were collected between 2007 and 2017. Moreover, the REVEAL 2.0 and REVEAL 2.0 Lite risk assessments were validated in our population. Overall, 370 PAH patients were included in the present study. Patients were predominantly female (78.5%) and had a mean age of 41.8 ± 18.8 years. Most patients (33.4%) had idiopathic PAH, 30.2% had PAH associated with congenital heart disease, and 23.5% had PAH associated with connective tissue disease. The low‐risk group showed significantly lower mortality than the intermediated‐ or high‐risk group at diagnosis (*p* < 0.05). In conclusion, our data suggest that REVEAL 2.0 and REVEAL 2.0 Lite risk assessments can predict mortality risk in PAH patients in Southern Brazil.

## INTRODUCTION

Pulmonary arterial hypertension (PAH) is characterized by pulmonary vascular obliteration and remodeling that leads to progressive right heart failure and death.^1^, ^2^ Last decades have witnessed significant improvements in knowledge about pathophysiology, epidemiology, prognostic factors, and treatment management; yet PAH remains a relentlessly progressive disease with unacceptable mortality.^3^ Most of the current epidemiological data derive from national European and US registries^4^, ^5^; these registries allowed not only a better understanding of the clinical aspects of the disease but also the development of risk stratification tools that nowadays represent the mainstem of PAH management.^6^, ^7^, ^8^, ^9^, ^10^, ^11^, ^12^

In the current PAH treatment guidelines, treatment is tailored according to the risk of disease progression and death.^13^ A combination of different risk variables defines well validated low‐, intermediate,‐ and high‐risk groups.^14^, ^15^, ^16^, ^17^ According to the risk classification stratum treatment is initiated from double oral combination therapy, for the low risk profile, to combination therapy including parenteral prostanoids, for the high risk patients, with the goal of keeping/bringing the patient to a low risk of progression.^18^, ^19^, ^20^

In this context, several risk scores are available to guide clinicians in determining prognosis for their patients with PAH, including The Registry to Evaluate Early and Long‐Term PAH Disease Management (REVEAL),^2^, ^14^, ^21^ REVEAL 2.0,^6^, ^22^ Comparative Prospective Registry of Newly Initiated Therapies for Pulmonary Hypertension (COMPERA),^23^, ^24^ Swedish PAH Registry (SPAHR),^15^, ^25^ and French Pulmonary Hypertension Network (FPHN).^9^ Generally, all have comparable efficacy in stratifying patients into low (<5%), intermediated (5%−10%), and high‐risk (>10%) of mortality at 1 year.^26^ Nevertheless, PAH patients from developing regions seem to have a slightly different clinical profile,^27^, ^28^, ^29^ raising the question about the potential bias on directly extrapolating these risk stratification strategies to these regions. Thus, the aim of the present study was to validate the risk assessment in a large cohort from Southern Brazil.

## METHODS

### The Southern Brazil PAH register

This was a multicentric retrospective cohort of prevalent cases, enrolling patients from nine pulmonary hypertension (PH) centers in Southern Brazil, including reference centers in the States of Rio Grande do Sul, Santa Catarina, and Paraná. The Registry of patients with Pulmonary Hypertension in Southern Brazil (RESPHIRAR) was created in January 2018 and was designed and fulfilled following Strengthening the Reporting of Observational Studies in Epidemiology (STROBE) statement.^30^, ^31^

A systematic data collection including baseline information of all consecutive PH patients diagnosed between 2007 and 2017 was performed. Date of the confirmatory right heart catheterization (RHC) was considered the date of diagnosis.^1^ PH was classified following the World Health Organization (WHO) guidelines.^32^ In RESPHIRAR registry, patients were eligible for enrollment if they met diagnostic criteria of PAH (group 1) or chronic thromboembolic pulmonary hypertension (CTEPH, group 4) according to international guidelines.^27^ Additionally, based on the etiology of PAH, patients were divided into subgroups. Recorded data included demographic characteristics, clinical and laboratorial parameters, WHO Functional Class (FC), hemodynamics, therapy usage, and survival status. The present study followed the ethical principles for medical research in humans from the Helsinki Declaration. The study protocol was registered at ensaiosclinicos.gov.br under the number RBR‐4fhbrp.

### Risk assessment

The REVEAL Lite 2.0 and the REVEAL 2.0 were calculated to provide a simplified method of mortality risk assessment for adult patients with PAH.^6^, ^22^ For the REVEAL Lite 2.0 assessment (with score ranging from 1 to 14), a score between 1 and 5 was considered low risk, a score of 6 or 7 was considered intermediated risk, and a score of 8 or higher was considered high risk.^22^ For the REVEAL 2.0 assessment (with scores ranging from 0 to 23), a score between 0 and 6 was considered low risk, a score of 7 or 8 was consider intermediate risk, and a score of 9 or higher was considered high risk.^6^

### Statistical analysis

Normal distribution of data was assessed using Kolmogorov−Smirnov test. Variables with normal distribution are presented as mean ± standard deviation. Variables with skewed distribution were log‐transformed before analysis and are presented as median (25th–75th percentiles). Categorical data are shown as percentages. One‐way ANOVA followed by Tukey post hoc tests was used to compare the continuous variables distribution among the three PAH subgroups. Values with significant differences were indicated by different superscript letters (*p* < 0.05), while statistically similar values were indicated by the same superscript letters. *χ* ^2^ test was used for categorical variables.

Survival analysis was performed using the Kaplan−Meier method, with the date of entry into the study defined as the date of the first diagnostic RHC. All‐cause mortality was defined as the endpoint and the log‐rank test was used for comparison between groups. Of note, Kaplan−Meier curves are absolute survival. None of the patients included in RESPHIRAR cohort underwent lung transplantation or atrial septostomy. The latter were censored at the time of their last evaluations. *p* < 0.05 were considered statistically significant. Statistical analyses were performed using the SPSS statistical package (v.28.0) for Windows (SPSS Inc.).

## RESULTS

### RESPHIRAR registry

The RESPHIRAR registry retrieved data from 602 PH prevalent patients. Of these, 67 patients had to be excluded because they were diagnosed with PH groups 2, 3, or 5. Additionally, another four patients were excluded because of missing data. Moreover, 61 were children or adolescents. A total of 470 adult patients were enrolled in the *RESPHIRAR* registry: most of them were female (77.3%), white (89.9%), and with a mean age of 48.3 ± 7.1 years. Among these 470 patients, 370 (78.7%) had PAH and were analyzed in great depth in the present article. Patient selection flowchart are shown in Supporting Information: Figure 1.

### PAH adult population description

**Figure 1:**
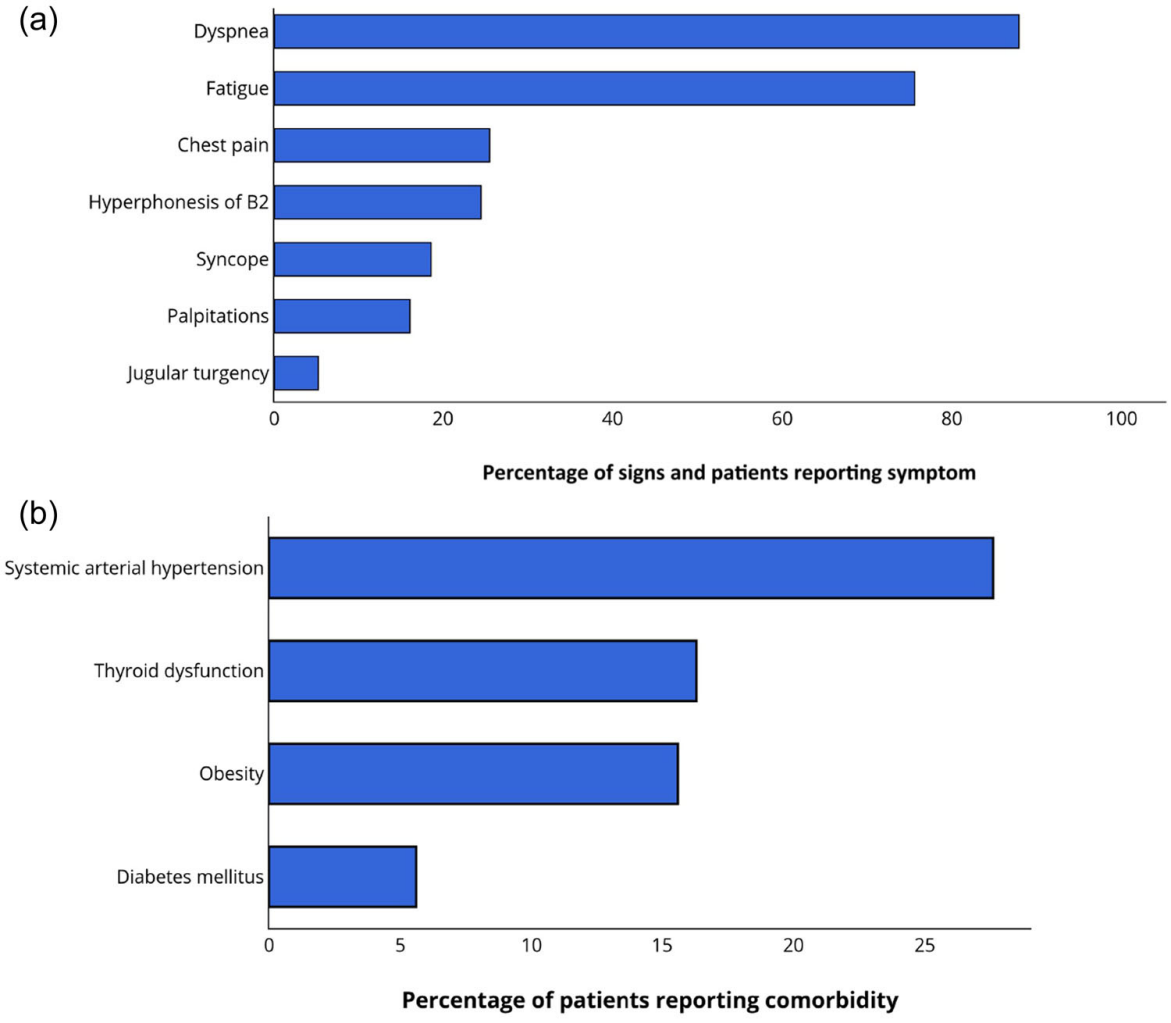
Characteristics of PAH sample at baseline. (a) Common signals and symptoms and (b) common comorbidities. PAH, pulmonary arterial hypertension.

Demographic, hemodynamic, and clinical characteristics at PAH diagnosis are presented in Table 1. Among of these patients, most of them female (78.5%), with mean age of 41.8 ± 18.8 years. Most of patients were in WHO FC II or III at diagnosis (80.2%). Most common treatment consisted of monotherapy in 72.3% of PAH patients, followed by double combination therapy in 26.3% and triple combination in 1.4%. The majority started with phosphodiesterase‐5 inhibitors (PDE5i) or endothelin‐receptor antagonist. At diagnosis, the most common presenting symptom was dyspnea (87.9%), followed by fatigue (78.1%) (Figure 1a). Moreover, at baseline, the most frequent comorbidity is system arterial hypertension (27%), followed by thyroid dysfunction, obesity, and diabetes mellitus (Figure 1b).

**Table 1:** Baseline clinical, laboratory, and hemodynamic characteristics of the study cohort by PAH‐type.

**Baseline characteristics**	**Overall PAH population (*n*** = **370)**	**Idiopathic PAH (*n*** = **125)**	**PAH‐CTD (*n*** = **88)**	**PAH‐CHD (*n*** = **116)**	***p* Value** *
Gender, *n* (% female)	295 (78.5)	101 (80.8)^a^	78 (88.6)^b^	83 (73.5)^a^	0.031
Age (years)	41.8 ± 18.8	45.1 ± 18.3^a^	54.4 ± 13.9^b^	37.9 ± 18.1^c^	<0.001
BMI (kg/m^2^)	25.2 ± 7.7	26.2 ± 10.3^a^	26.5 ± 6.9^a^	22.8 ± 4.9^b^	0.004
WHO functional class, *n* (%)					
I	38 (10.3)	7 (5.6)	10 (11.4)	17 (15.0)	0.080
II	157 (42.4)	48 (38.4)	37 (42.0)	53 (46.9)	
III	140 (37.8)	56 (44.8)	31 (35.2)	33 (29.2)	
IV	35 (9.5)	14 (11.2)	9 (10.2)	8 (7.1)	
6 min walk distance (m)	403.1 ± 124.6	413.2 ± 119.2	367.8 ± 114.0	400.9 ± 133.6	0.054
NT‐proBNP (pg/ml)**	366 (124−1278)	543 (103−2010)	259 (129−533)	460 (213−1768)	0.375
Hemodynamic data at diagnosis					
Systolic PAP (mmHg)	80.2 ± 26.9	82.2 ± 24.7^a^	67.3 ± 24.6^b^	87.3 ± 28.4^a^	<0.001
Diastolic PAP (mmHg)	37.1 ± 15.2	37.9 ± 13.7^a^	29.1 ± 11.3^b^	41.3 ± 16.5^c^	<0.001
mPAP (mmHg)	51.8 ± 17.9	53.7 ± 16.3^a^	41.8 ± 14.2^b^	57.8 ± 19.9^c^	<0.001
PAWP (mmHg)	10.0 (8.0 – 12.0)	10.2 ± 4.4	10.4 ± 4.1	10.1 ± 3.6	0.873
PVR (Woods)	7.9 (4.8−11.9)	9.3 (5.3−12.2)	7.0 (3.6−10.0)	9.4 (5.5−13.0)	0.294
Cardiac index (L/min)	3.2 ± 1.4	2.7 ± 0.9^a^	3.2 ± 1.1^a,b^	3.6 ± 1.5^b^	0.002
Cardiac output (L/min)	5.2 ± 2.1	5.2 ± 1.9	5.0 ± 2.1	5.6 ± 2.1	0.307
PAH‐specific therapy (%)					
Monotherapy	268 (72.4)	88 (70.4)	62 (70.5)	86 (74.1)	0.652
Dual therapy	97 (26.3)	34 (27.2)	25 (28.4)	29 (25.0)	
Triple therapy	5 (1.4)	3 (2.4)	1 (1.1)	1 (0.9)	

*Note*: Results are presented as mean ± SD, *n* (%), or median (25th−75th), as appropriated.

Abbreviations: BMI, body mass index; mPAP, mean pulmonary artery pressure; PAH, pulmonary arterial hypertension; PAH‐CHD, PAH associated with congenital heart disease; PAH‐CTD, PAH associated with connective tissue disease; PAP, pulmonary artery pressure; PAWP, pulmonary arterial wedge pressure; PVR, pulmonary vascular resistance; SD, standard deviation; WHO, world health organization.

* *p* Value*s* were computed using *χ* ^2^ or ANOVA, followed by post hoc multiple comparison tests (residual analysis or Tukey's tests, respectively), as appropriate. Values with significant differences are indicated by different superscript letters (*p* < 0.05), while statistically similar values are indicated by the same superscript letters.

** Data available for 260 patients.

### Subgroups of PAH

The most frequently diagnosed type of PAH was idiopathic (33.4%), followed by PAH associated with congenital heart disease (PAH‐CHD, 30.2%), and PAH associated with connective tissue disease (PAH‐CTD, 23.5%). The other causes contribute for less than 5% each, including HIV (4.8%), portopulmonary hypertension (4.0%), hereditary (1.4%), veno‐occlusive (1.6%), and drug‐induced PAH (1.1%).

The distribution and characteristics of patients with idiopathic PAH, PAH‐CHD, or PAH‐CTD are shown in Table 1. The three groups are different in age. As expected, patients with PAH‐CHD were younger and presented lower BMI levels compared to idiopathic and PAH‐CTD subgroups, while patients with PAH‐CTD were older than the other two groups. The prevalence of women was higher in PAH‐CTD group compared to the other two groups. Although the significance did not reach formal statistical levels, PAH‐CTD patients walked less than the other two groups (*p* > 0.05). Considering hemodynamic data, the three subgroups differ in terms of systolic and diastolic PAP and mPAP measurements (*p* < 0.001).

Of note, among PAH‐CTD patients, 63.9% had systemic sclerosis, 11.4% had lupus, 20.2% had other collagen disease, and 4.5% had Sjogren. Of the PAH‐CHD patients, most of patients had atrial septal defect (37.9%) or ventricular septal defect (28.4%).

### Survival analysis

**Figure 2:**
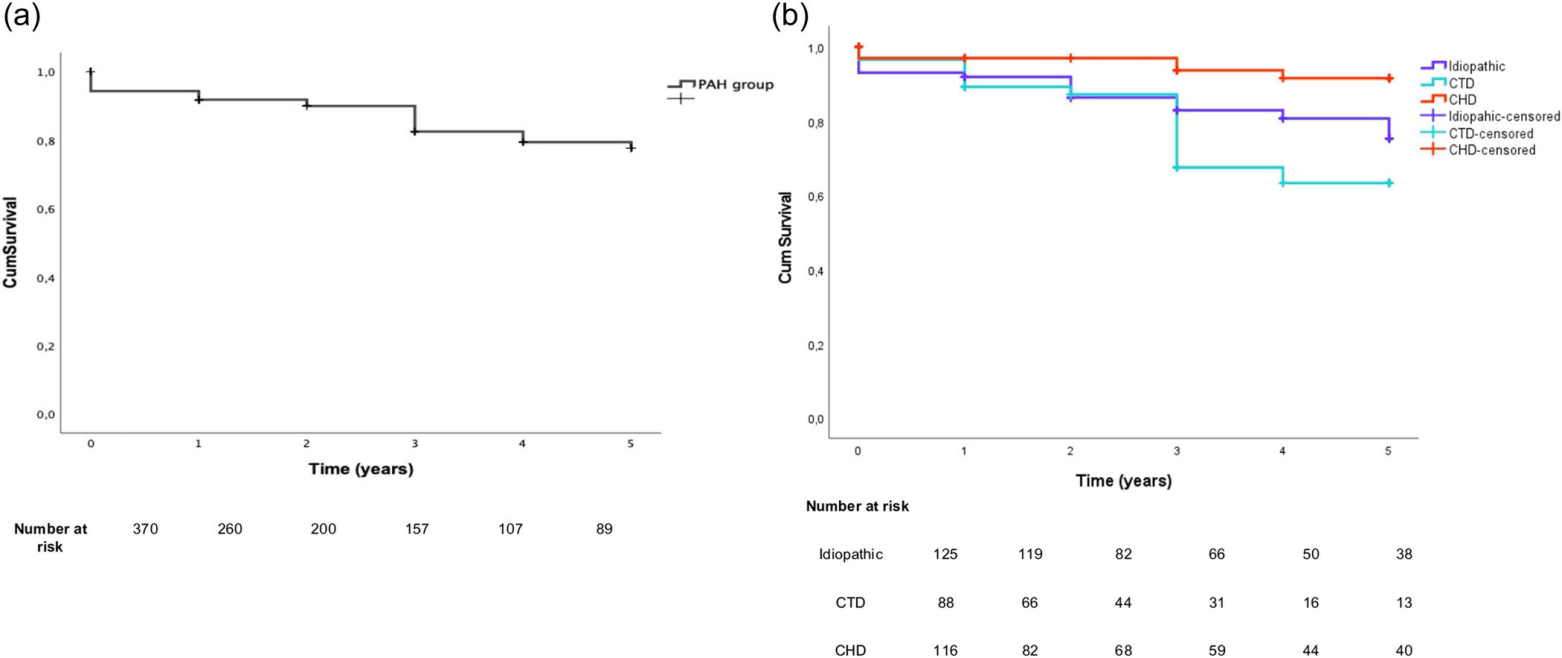
Kaplan−Meier estimated of 5‐year survival from data of diagnosis in PAH patients. (a) Overall PAH population. (b) PAH subgroups. PAH, pulmonary arterial hypertension.

Median follow‐up period was 36 months (14–70 months) for PAH. Survival rates at 1, 3, and 5 years were 92.4%, 83.1%, and 78.2% for PAH patients. The Kaplan−Meier curve for both patient groups is illustrated in Figure 2a.

Kaplan–Meier survival curves according to different PAH subgroups etiologies are shown in Figure 2b. PAH‐CTD patients presented the worst survival (4.0 [95% CI: 3.6–4.4 years], followed by idiopathic (4.4 [95% CI: 4.1–4.7 years]) and PAH‐CHD (4.8 [95% CI: 4.3–5.0]). Survival rates at 5 years were 76.6% for idiopathic PAH patients, 63.2% for PAH‐CTD patients, and 91.5% for PAH‐CHD patients (p = 0.003; Figure 2b).

### Risk assessment at time of PAH diagnosis

**Figure 3:**
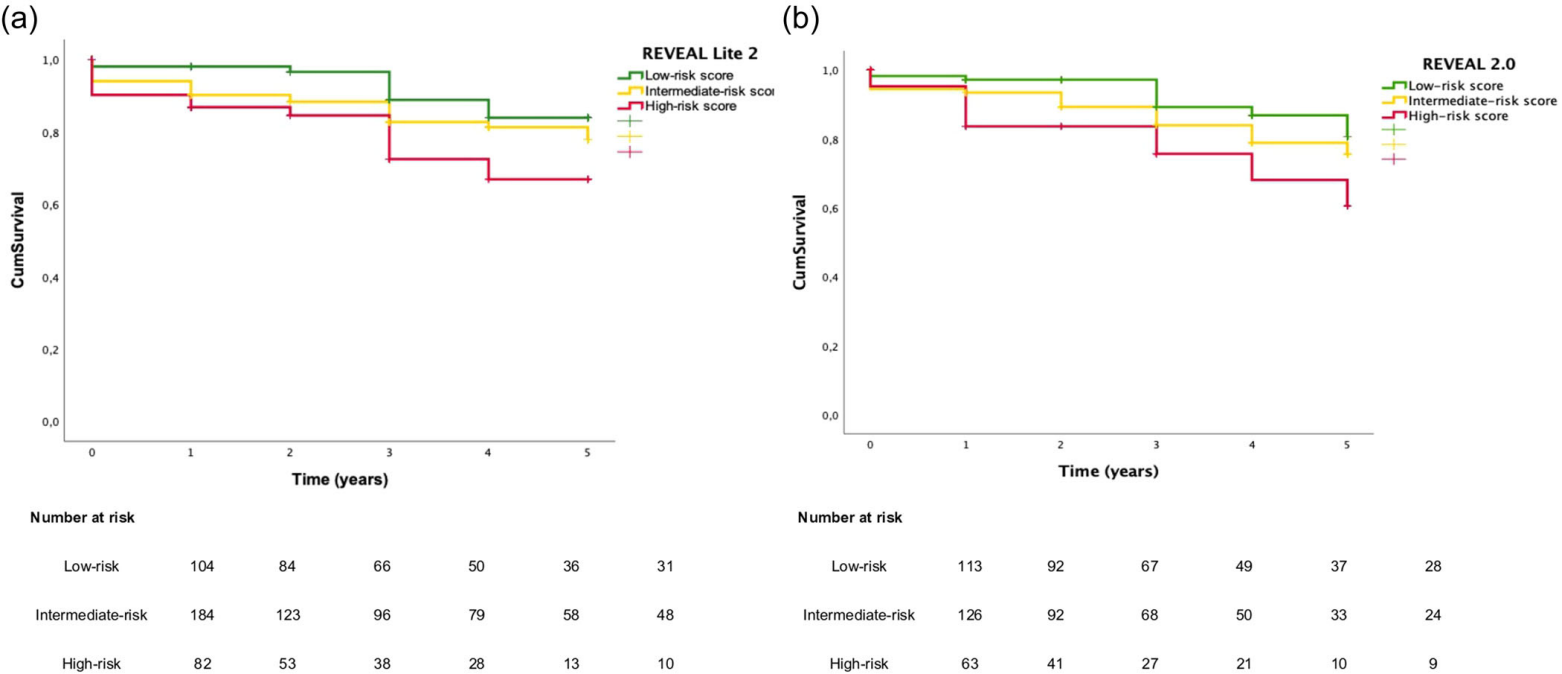
Kaplan−Meier estimated of 5‐year survival from data of diagnosis in PAH patients. (a) REVEAL Lite 2.0. (b) REVEAL 2.0. PAH, pulmonary arterial hypertension.

At baseline, the mean REVEAL Lite 2.0 risk score was 6.3 ± 1.6 for PAH patients. Most patients (49.7%) were in the intermediate‐risk stratum at baseline, 28.1% were in the low‐risk stratum, and 22.2% were in the high‐risk stratum. After 5‐years of follow‐up, survival of patients at low‐risk at baseline was 84.7%, at intermediate‐risk was 78.4%, and at high‐risk was 67.5% (Log‐rank p = 0.021, Figure 3a). In the same way, implementing the REVEAL 2.0 risk score the similar results were found: 41.7% of patients were in the intermediate‐risk stratum at baseline, 37.4% were in the low‐risk stratum, and 20.9% were in the high‐risk stratum. After 5‐years of follow‐up, survival of patients at low‐risk at baseline was 81.7%, at intermediate‐risk was 76.4%, and at high‐risk was 60.5% (Log‐rank p = 0.034, Figure 3b). Interestingly, when comparing low risk to intermediate + high‐risk, the REVEAL 2.0, this stratification score was able to differentiate the two groups regarding the survival of patients at 5‐years of follow‐up (*p* = 0.034). For both stratifications risk scores, there is no difference among PAH subgroups (*p* > 0.05).

## DISCUSSION

In the present study, we retrospectively validated the REVEAL 2.0 and REVEAL Lite 2.0 also for developing regions, with specific patients' characteristics. Using these two‐risk assessment, we demonstrated that patients in the low‐risk group at baseline had significantly low mortality than those in the intermediate‐ and high‐risk groups in a cohort of PAH patients from Southern Brazil.

Generally, patients with PAH in our registry were comparable to those in other registries in terms of WHO FC distribution, female‐to‐male ratio, main hemodynamics parameters, and symptoms at diagnosis. However, the most interestingly finding is that the mean 6 min walking distance (6MWD) in participants of our cohort was higher compared to several registries.^1^, ^27^, ^33^, ^34^ This result agrees with previous data other Brazilian cohorts that has also suggested that the 6MWD is higher than the one found in United States and Europe.^1^, ^7^ One of possible explanations for these findings is that the mean age of patients was lower in Brazil, which could contribute to a better 6MWD.^27^, ^28^

Although registries from US and Europe had described an increasing proportion of older patients diagnosed with idiopathic PAH, in developing countries, the average age of patients with idiopathic PAH is younger than 40 years,^27^, ^28^, ^29^, ^35^ which corroborate with the RESPHIRAR registry findings. These differences may be explained by several factors, including overall population age distribution between developed and developing countries (older population in Europe and United States) and healthcare systems.^35^, ^36^ However, other factors may play a role such as: referral patterns, PAH awareness, increase patient access to information, and widespread use of noninvasive screening tools.^1^

Corroborating with this result, the percentage of CHD‐PAH patients in our population was also higher compared to the frequency described in most registries, especially the ones from European population.^33^, ^37^, ^38^ It is known that developing countries have a greater number of patients with uncorrected or late correction congenital heart disease in childhood. Thus, it is vitally important to have a global view of PAH, as registries in developing countries may be exhibit a different distribution of disease pattern.^27^

These differences in mortality rate may be a result of ethnicity or national differences between the populations in Brazil and Europe, such as the characteristics of CHD‐PAH.^39^, ^40^, ^41^ It is noteworthy that the proportion of CHD‐PAH patients reported in our study is similar to Argentina,^42^ but higher compared to Europe^37^, ^38^ and North America,^43^, ^44^ which could explain why we have a higher survival rate than in other studies at 1, 3, or 5 years of follow‐up, even though we have fewer patients on double and triple combination therapy.

Patients with PAH‐CHD had the best survival in the PAH group, which agrees with previous publications that have showed good long‐term survival in these patients.^9^, ^33^, ^38^, ^45^ In a study published by Manes et al., the 5‐year survival of patients with PAH‐CHD was 91% compared with 63% in the contemporary group of idiopathic PAH patients in which the same treatment strategy was used.^46^ In other series, a 3‐year survival rates of 70% for idiopathic, heritable, or anorexigenic‐associated PAH^47^ and 56% for PAH‐CTD^48^ was described.

In the same line, a previous published study including incident cases from Brazil have shown that the third leading cause of PAH following idiopathic PAH and CTD‐PAH was schistosomiasis (Sch–PAH).^28^ According to the National Survey on the Prevalence of *Schistosomiasis mansoni* and *Geohelminthiasis*,^49^ we can verify that there is a large discrepancy in the prevalence of schistosomiasis between different regions of Brazil. The southern region of Brazil is not an endemic region for schistosomiasis, and this may be an explanation for why no cases with this pathology were found in RESPHIRAR population.

Considering that there is no single variable that predicts outcomes in PAH patients, risk assessment in PAH patients should include a range of clinical, hemodynamic, and exercise parameters performed in a serial fashion to reflect a patient's course during the disease.^13^, ^50^ Risk stratification is an essential and relatively simple process to inform clinicians about prognosis, select treatment options, objectively monitor treatment response or disease progression, and optimize the timing of certain interventions such as lung transplantation.^20^ The predominance of the intermediate risk group at baseline and the worse outcome as compared with the low‐risk group has also been observed in the other registries studies.^15^, ^16^, ^17^

Risk assessment is especially important in settings where clinical PAH experience is not available, as it could facilitate timely referral to a PAH center and/or lung transplantation.^50^ In this context, REVEAL Lite 2.0 has clinical usefulness in screening patients because it can be used as a relatively quick and simple method for accurately identifying patients predicted to have a low risk of mortality. The REVEAL risk score calculator and its updated version (REVEAL 2.0) appeared accurate and well calibrated in validation cohorts, which confirms their generalizability.^6^

Registries are important to characterize populations, assess the burden of diseases and describe practice patterns. In contrast to a prospective study, registries generally do not provide scheduled data entry or allow inclusion of patients only according to predefined datasets.^1^, ^5^ Patient registries collecting observational data can be of great value in the understanding of clinical problems. While clinical trials provide data in selected patient populations, registries better depict real‐life practice.^5^

Some strengths and limitations of the present study should be acknowledged. Among the study's strengths are the sample size, being a multicenter study with standardized, albeit retrospective, data collection, and comparable results. As limitations, first, the potential loss of patients to follow‐up and the difficulty defining the time of diagnosis in prevalent patients. The study is prone to common standard limitations of a register‐based descriptive study, including (1) lack of a standardized treatment protocol; (2) immortal time bias, which refers to a span of time in the observation or follow‐up period of a cohort during which the outcome under study could not have occurred; and (3) case‐wise deletion of patients due to missing data.

In conclusion, we found that the risk assessment strategy proposed by REVEAL 2.0 and REVEAL Lite 2.0 is valid for the prediction of long‐term prognosis in PAH patients undergoing treatment in Brazil. Importantly, objective multivariable risk scores are better at predicting a patient's risk than clinical gestalt which is why such tools are essential in modern clinical practice.

## AUTHOR CONTRIBUTIONS

All authors have participated in the conceptualization, writing and interpretation of the content and have approved the final version of this article.

## CONFLICTS OF INTEREST STATEMENT

Gláucia Maria Barbieri reports speaker and sponsorships in scientific events by Janssen and Bayer. Ariovaldo Leal Fagundes reports lectures to GlaxoSmithKline, Pfizer, and AstraZeneca. Scientific consultancy fee from Ache, Boehringer Ingelheim, and Chiesi. Roger Pirath Rodrigues reports lectures to Janssen, Bayer, Astra‐Zeneca, GSK. Rogerio Souza reports lecture and consultancy fees from Acceleron, Bayer, and Merck. Gisela Martina Bohns Meyer reports lecture and consultancy fees from Aceeleron, Bayer, and GSK. The remaining authors declare no conflict of interest. Conflict of interest outside the submitted work.

## ETHICS STATEMENT

The study was approved by an institutional review board.

## Supporting information

Figurementary figure 1.

## References

[1] McGoon MD , Benza RL , Escribano‐Subias P , Jiang X , Miller DP , Peacock AJ , Pepke‐Zaba J , Pulido T , Rich S , Rosenkranz S , Suissa S , Humbert M . Pulmonary arterial hypertension. JACC. 2013;62(25 suppl):D51–9. 24355642 10.1016/j.jacc.2013.10.023

[2] Farber HW , Miller DP , Poms AD , Badesch DB , Frost AE , Rouzic EML , Romero AJ , Benton WW , Elliott CG , McGoon MD , Benza RL . Five‐year outcomes of patients enrolled in the REVEAL registry. Chest. 2015;148(4):1043–54. 26066077 10.1378/chest.15-0300

[3] Deshwal H , Weinstein T , Sulica R . Advances in the management of pulmonary arterial hypertension. J Investig Med. 2021;69(7):1270–80. 10.1136/jim-2021-002027 PMC8485135 34580123

[4] Humbert M . Lessons from pulmonary hypertension registries. Rev Port Cardiol. 2018;37(9):759–61. 30143347 10.1016/j.repc.2018.08.003

[5] Swinnen K , Quarck R , Godinas L , Belge C , Delcroix M . Learning from registries in pulmonary arterial hypertension: pitfalls and recommendations. Eur Respir Rev. 2019;28(154):190050. 31852746 10.1183/16000617.0050-2019 PMC9488628

[6] Benza RL , Gomberg‐Maitland M , Elliott CG , Farber HW , Foreman AJ , Frost AE , McGoon MD , Pasta DJ , Selej M , Burger CD , Frantz RP . Predicting survival in patients with pulmonary arterial hypertension. Chest. 2019;156(2):323–37. 30772387 10.1016/j.chest.2019.02.004

[7] Benza RL , Miller DP , Gomberg‐Maitland M , Frantz RP , Foreman AJ , Coffey CS , Frost A , Barst RJ , Badesch DB , Elliott CG , Liou TG , McGoon MD . Predicting survival in pulmonary arterial hypertension: insights from the registry to evaluate early and long‐term pulmonary arterial hypertension disease management (REVEAL). Circulation. 2010;122(2):164–72. 20585012 10.1161/CIRCULATIONAHA.109.898122

[8] D'Alonzo GE , Barst RJ , Ayres SM , Bergofsky EH , Brundage BH , Detre KM , Fishman AP , Goldring RM , Groves BM , Kernis JT . Survival in patients with primary pulmonary hypertension. Results from a national prospective registry. Ann Intern Med. 1991;115(5):343–9. 1863023 10.7326/0003-4819-115-5-343

[9] Humbert M , Sitbon O , Yaici A , Montani D , O'Callaghan DS , Jais X , Parent F , Savale L , Natali D , Gunther S , Chaouat A , Chabot F , Cordier JF , Habib G , Gressin V , Jing ZC , Souza R , Simonneau G . Survival in incident and prevalent cohorts of patients with pulmonary arterial hypertension. Eur Respir J. 2010;36(3):549–55. 20562126 10.1183/09031936.00057010

[10] Ling Y , Johnson MK , Kiely DG , Condliffe R , Elliot CA , Gibbs JSR , Howard LS , Pepke‐Zaba J , Sheares KKK , Corris PA , Fisher AJ , Lordan JL , Gaine S , Coghlan JG , Wort SJ , Gatzoulis MA , Peacock AJ . Changing demographics, epidemiology, and survival of incident pulmonary arterial hypertension: results from the pulmonary hypertension registry of the United Kingdom and Ireland. Am J Respir Crit Care Med. 2012;186(8):790–6. 22798320 10.1164/rccm.201203-0383OC

[11] Thenappan T , Glassner C , Gomberg‐Maitland M . Validation of the pulmonary hypertension connection equation for survival prediction in pulmonary arterial hypertension. Chest. 2012;141(3):642–50. 21885728 10.1378/chest.11-0969

[12] Sitbon O , Benza RL , Badesch DB , Barst RJ , Elliott CG , Gressin V , Lemarié JC , Miller DP , Muros‐Le Rouzic E , Simonneau G , Frost AE , Farber HW , Humbert M , McGoon MD . Validation of two predictive models for survival in pulmonary arterial hypertension. Eur Respir J. 2015;46(1):152–64. 25837032 10.1183/09031936.00004414

[13] Galiè N , Humbert M , Vachiery JL , Gibbs S , Lang I , Torbicki A , Simonneau G , Peacock A , Vonk Noordegraaf A , Beghetti M , Ghofrani A , Gomez Sanchez MA , Hansmann G , Klepetko W , Lancellotti P , Matucci M , McDonagh T , Pierard LA , Trindade PT , Zompatori M , Hoeper M , ESC Scientific Document Group . 2015 ESC/ERS guidelines for the diagnosis and treatment of pulmonary hypertension: the joint task force for the diagnosis and treatment of pulmonary hypertension of the European Society of Cardiology (ESC) and the European Respiratory Society (ERS): endorsed by: Association for European Paediatric and Congenital Cardiology (AEPC), International Society for Heart and Lung Transplantation (ISHLT). Eur Heart J. 2016;37(1):67–119. 26320113 10.1093/eurheartj/ehv317

[14] Benza RL , Gomberg‐Maitland M , Miller DP , Frost A , Frantz RP , Foreman AJ , Badesch DB , McGoon MD . The REVEAL registry risk score calculator in patients newly diagnosed with pulmonary arterial hypertension. Chest. 2012;141(2):354–62. 21680644 10.1378/chest.11-0676

[15] Kylhammar D , Kjellström B , Hjalmarsson C , Jansson K , Nisell M , Söderberg S , Wikström G , Rådegran G . A comprehensive risk stratification at early follow‐up determines prognosis in pulmonary arterial hypertension. Eur Heart J. 2018;39(47):4175–81. 28575277 10.1093/eurheartj/ehx257

[16] Hoeper MM , Kramer T , Pan Z , Eichstaedt CA , Spiesshoefer J , Benjamin N , Olsson KM , Meyer K , Vizza CD , Vonk‐Noordegraaf A , Distler O , Opitz C , Gibbs JSR , Delcroix M , Ghofrani HA , Huscher D , Pittrow D , Rosenkranz S , Grünig E . Mortality in pulmonary arterial hypertension: prediction by the 2015 European pulmonary hypertension guidelines risk stratification model. Eur Respir J. 2017;50(2):1700740. 28775047 10.1183/13993003.00740-2017

[17] Boucly A , Weatherald J , Savale L , Jaïs X , Cottin V , Prevot G , Picard F , de Groote P , Jevnikar M , Bergot E , Chaouat A , Chabanne C , Bourdin A , Parent F , Montani D , Simonneau G , Humbert M , Sitbon O . Risk assessment, prognosis and guideline implementation in pulmonary arterial hypertension. Eur Respir J. 2017;50(2):1700889. 28775050 10.1183/13993003.00889-2017

[18] Weatherald J , Boucly A , Sahay S , Humbert M , Sitbon O . The low‐risk profile in pulmonary arterial hypertension. Time for a paradigm shift to goal‐oriented clinical trial endpoints? Am J Respir Crit Care Med. 2018;197(7):860–8. 29256625 10.1164/rccm.201709-1840PP

[19] Galiè N , Channick RN , Frantz RP , Grünig E , Jing ZC , Moiseeva O , Preston IR , Pulido T , Safdar Z , Tamura Y , McLaughlin VV . Risk stratification and medical therapy of pulmonary arterial hypertension. Eur Respir J. 2019;53(1):1801889. 30545971 10.1183/13993003.01889-2018 PMC6351343

[20] Kjellström B , Hjalmarsson C , Kylhammar D , Rådegran G . Pulmonary arterial hypertension: assessing risk to improve prognosis. Expert Rev Cardiovasc Ther. 2019;17(1):1–2. 30422719 10.1080/14779072.2019.1548278

[21] Benza RL , Miller DP , Foreman AJ , Frost AE , Badesch DB , Benton WW , McGoon MD . Prognostic implications of serial risk score assessments in patients with pulmonary arterial hypertension: a registry to evaluate early and long‐term pulmonary arterial hypertension disease management (REVEAL) analysis. J Heart Lung Transplant. 2015;34(3):356–61. 25447572 10.1016/j.healun.2014.09.016

[22] Benza RL , Kanwar MK , Raina A , Scott JV , Zhao CL , Selej M , Elliott CG , Farber HW . Development and validation of an abridged version of the REVEAL 2.0 risk score calculator, REVEAL lite 2, for use in patients with pulmonary arterial hypertension. Chest. 2021;159(1):337–46. 32882243 10.1016/j.chest.2020.08.2069 PMC7462639

[23] Hoeper MM , Huscher D , Ghofrani HA , Delcroix M , Distler O , Schweiger C , Grunig E , Staehler G , Rosenkranz S , Halank M , Held M , Grohé C , Lange TJ , Behr J , Klose H , Wilkens H , Filusch A , Germann M , Ewert R , Seyfarth HJ , Olsson KM , Opitz CF , Gaine SP , Vizza CD , Vonk‐Noordegraaf A , Kaemmerer H , Gibbs JSR , Pittrow D . Elderly patients diagnosed with idiopathic pulmonary arterial hypertension: results from the COMPERA registry. Int J Cardiol. 2013;168(2):871–80. 23164592 10.1016/j.ijcard.2012.10.026

[24] Hoeper MM , Kramer T , Pan Z , Eichstaedt CA , Spiesshoefer J , Benjamin N , Olsson KM , Meyer K , Vizza CD , Vonk‐Noordegraaf A , Distler O , Opitz C , Gibbs JSR , Delcroix M , Ghofrani HA , Huscher D , Pittrow D , Rosenkranz S , Grünig E . Mortality in pulmonary arterial hypertension: prediction by the 2015 European pulmonary hypertension guidelines risk stratification model. Eur Respir J. 2017;50(2):1700740. 28775047 10.1183/13993003.00740-2017

[25] Rådegran G , Kjellström B , Ekmehag B , Larsen F , Rundqvist B , Blomquist SB , Gustafsson C , Hesselstrand R , Karlsson M , Kornhall B , Nisell M , Persson L , Ryftenius H , Selin M , Ullman B , Wall K , Wikström G , Willehadson M , Jansson K . Characteristics and survival of adult Swedish PAH and CTEPH patients 2000−2014. Scand Cardiovasc J. 2016;50(4):243–50. 27146648 10.1080/14017431.2016.1185532

[26] Thomas CA , Anderson RJ , Condon DF , de Jesus Perez VA . Diagnosis and management of pulmonary hypertension in the modern era: insights from the 6th world symposium. Pulmonary Therapy. 2020;6(1):9–22. 32048239 10.1007/s41030-019-00105-5 PMC7229067

[27] Valverde AB , Soares JM , Viana KP , Gomes B , Soares C , Souza R . Pulmonary arterial hypertension in Latin America: epidemiological data from local studies. BMC Pulm Med. 2018;18(1):106. 29940945 10.1186/s12890-018-0667-8 PMC6019295

[28] Alves JL , Gavilanes F , Jardim C , Fernandes CJCS , Morinaga LTK , Dias B , Hoette S , Humbert M , Souza R . Pulmonary arterial hypertension in the Southern Hemisphere. Chest. 2015;147(2):495–501. 25317567 10.1378/chest.14-1036

[29] Jing ZC , Xu XQ , Han ZY , Wu Y , Deng KW , Wang H , Wang ZW , Cheng XS , Xu B , Hu SS , Hui RT , Yang YJ . Registry and survival study in Chinese patients with idiopathic and familial pulmonary arterial hypertension. Chest. 2007;132(2):373–9. 17400671 10.1378/chest.06-2913

[30] Vandenbroucke JP , von Elm E , Altman DG , Gøtzsche PC , Mulrow CD , Pocock SJ , Poole C , Schlesselman JJ , Egger M . Strengthening the reporting of observational studies in epidemiology (STROBE): explanation and elaboration. Int J Surg. 2014;12(12):1500–24. 25046751 10.1016/j.ijsu.2014.07.014

[31] von Elm E , Altman DG , Egger M , Pocock SJ , Gøtzsche PC , Vandenbroucke JP . The strengthening the reporting of observational studies in epidemiology (STROBE) statement: guidelines for reporting observational studies. JCE. 2008;61(4):344–9. 18313558 10.1016/j.jclinepi.2007.11.008

[32] Simonneau G , Montani D , Celermajer DS , Denton CP , Gatzoulis MA , Krowka M , Williams PG , Souza R . Haemodynamic definitions and updated clinical classification of pulmonary hypertension. Eur Respir J. 2019;53(1):1801913. 30545968 10.1183/13993003.01913-2018 PMC6351336

[33] Gall H , Felix JF , Schneck FK , Milger K , Sommer N , Voswinckel R , Franco OH , Hofman A , Schermuly RT , Weissmann N , Grimminger F , Seeger W , Ghofrani HA . The Giessen pulmonary hypertension registry: survival in pulmonary hypertension subgroups. J Heart Lung Transplant. 2017;36(9):957–67. 28302503 10.1016/j.healun.2017.02.016

[34] Escribano‐Subias P , Blanco I , López‐Meseguer M , Lopez‐Guarch CJ , Roman A , Morales P , Castillo‐Palma MJ , Segovia J , Gómez‐Sanchez MA , Barberà JA . Survival in pulmonary hypertension in Spain: insights from the Spanish registry. Eur Respir J. 2012;40(3):596–603. 22362843 10.1183/09031936.00101211

[35] Hoeper MM , Humbert M , Souza R , Idrees M , Kawut SM , Sliwa‐Hahnle K , Jing ZC , Gibbs JSR . A global view of pulmonary hypertension. Lancet Resp Med. 2016;4(4):306–22. 10.1016/S2213-2600(15)00543-3 26975810

[36] Hoeper MM , Simon R. Gibbs J . The changing landscape of pulmonary arterial hypertension and implications for patient care. Eur Respir Rev. 2014;23(134):450–7. 25445943 10.1183/09059180.00007814 PMC9487398

[37] Humbert M , Sitbon O , Chaouat A , Bertocchi M , Habib G , Gressin V , Yaici A , Weitzenblum E , Cordier JF , Chabot F , Dromer C , Pison C , Reynaud‐Gaubert M , Haloun A , Laurent M , Hachulla E , Simonneau G . Pulmonary arterial hypertension in France: results from a national registry. Am J Respir Crit Care Med. 2006;173(9):1023–30. 16456139 10.1164/rccm.200510-1668OC

[38] Alonso‐Gonzalez R , Lopez‐Guarch CJ , Subirana‐Domenech MT , Ruíz JMO , González IO , Cubero JS , del Cerro MJ , Salvador ML , Subira LD , Gallego P , Escribano‐Subias P . Pulmonary hypertension and congenital heart disease: an insight from the REHAP National Registry. Int J Cardiol. 2015;184:717–23. 25781723 10.1016/j.ijcard.2015.02.031

[39] Condliffe R , Clift P , Dimopoulos K , Tulloh RMR . Management dilemmas in pulmonary arterial hypertension associated with congenital heart disease. Pulm Circ. 2018;8(3):1–12. 10.1177/2045894018792501 PMC6161209 30033821

[40] D'Alto M , Mahadevan VS . Pulmonary arterial hypertension associated with congenital heart disease. Eur Respir Rev. 2012;21(126):328–37. 23204121 10.1183/09059180.00004712 PMC9487226

[41] Maurer SJ , Stöckemann K , Pujol C , Hörer J , Ewert P , Tutarel O . Pulmonary arterial hypertension associated with congenital heart disease in adults over the age of 40 years. J Clin Med. 2020;9(12):4071. 33348628 10.3390/jcm9124071 PMC7766787

[42] ML T , JO C , LE F , F K , RP B , GE B . Hipertensión arterial pulmonar: Registro de un centro de referencia em Argentina. Rev Am Med Respir. 2014;14:144–52.

[43] Badlam JB , Badesch DB , Austin ED , Benza RL , Chung WK , Farber HW , Feldkircher K , Frost AE , Poms AD , Lutz KA , Pauciulo MW , Yu C , Nichols WC , Elliott CG , Simms R , Fortin T , Safdar Z , Burger CD , Frantz RP , Hill NS , Airhart S , Elwing J , Simon M , White RJ , Robbins IM , Chakinala MM . United States pulmonary hypertension scientific registry. Chest. 2021;159(1):311–27. 32858008 10.1016/j.chest.2020.07.088 PMC7803940

[44] Badesch DB , Raskob GE , Elliott CG , Krichman AM , Farber HW , Frost AE , Barst RJ , Benza RL , Liou TG , Turner M , Giles S , Feldkircher K , Miller DP , McGoon MD . Pulmonary arterial hypertension. Chest. 2010;137(2):376–87. 19837821 10.1378/chest.09-1140

[45] Hurdman J , Condliffe R , Elliot CA , Davies C , Hill C , Wild JM , Capener D , Sephton P , Hamilton N , Armstrong IJ , Billings C , Lawrie A , Sabroe I , Akil M , O'Toole L , Kiely DG . ASPIRE registry: assessing the spectrum of pulmonary hypertension identified at a REferral centre. Eur Respir J. 2012;39(4):945–55. 21885399 10.1183/09031936.00078411

[46] Manes A , Palazzini M , Leci E , Bacchi Reggiani ML , Branzi A , Galie N . Current era survival of patients with pulmonary arterial hypertension associated with congenital heart disease: a comparison between clinical subgroups. Eur Heart J. 2014;35(11):716–24. 23455361 10.1093/eurheartj/eht072

[47] Humbert M , Sitbon O , Chaouat A , Bertocchi M , Habib G , Gressin V , Yaïci A , Weitzenblum E , Cordier J , Chabot F , Dromer C , Pison C , Reynaud‐Gaubert M , Haloun A , Laurent M , Hachulla E , Cottin V , Degano B , Jaïs X , Montani D , Souza R , Simonneau G . Survival in patients with idiopathic, familial, and anorexigen‐associated pulmonary arterial hypertension in the modern management era. Circulation. 2010;122(2):156–63. 20585011 10.1161/CIRCULATIONAHA.109.911818

[48] Hachulla E , Carpentier P , Gressin V , Diot E , Allanore Y , Sibilia J , Launay D , Mouthon L , Jego P , Cabane J , de Groote P , Chabrol A , Lazareth I , Guillevin L , Clerson P , Humbert M , ItinérAIR‐Sclérodermie Study Investigators . Risk factors for death and the 3‐year survival of patients with systemic sclerosis: the French ItinérAIR‐Sclérodermie study. Rheumatology (Oxford). 2009;48(3):304–8. 19174571 10.1093/rheumatology/ken488 PMC2644045

[49] Katz N. Inquérito Nacional de Prevalência da Esquistossomose mansoni e Geo‐helmintoses: FioCruz. 2018;1−90.

[50] Benza RL , Farber HW , Selej M , Gomberg‐Maitland M . Assessing risk in pulmonary arterial hypertension: what we know, what we don't. Eur Respir J. 2017;50(2):1701353. 28775053 10.1183/13993003.01353-2017

