# Supplementary figures and images for: Risk assessment validation in patients with pulmonary arterial hypertension: Data from a Southern Brazil registry (RESPHIRAR study)
Source: Pulm Circ. 2023 Jan 1;13(1):e12193. doi: 10.1002/pul2.12193 (PMC10031811; doi:10.1002/pul2.12193)

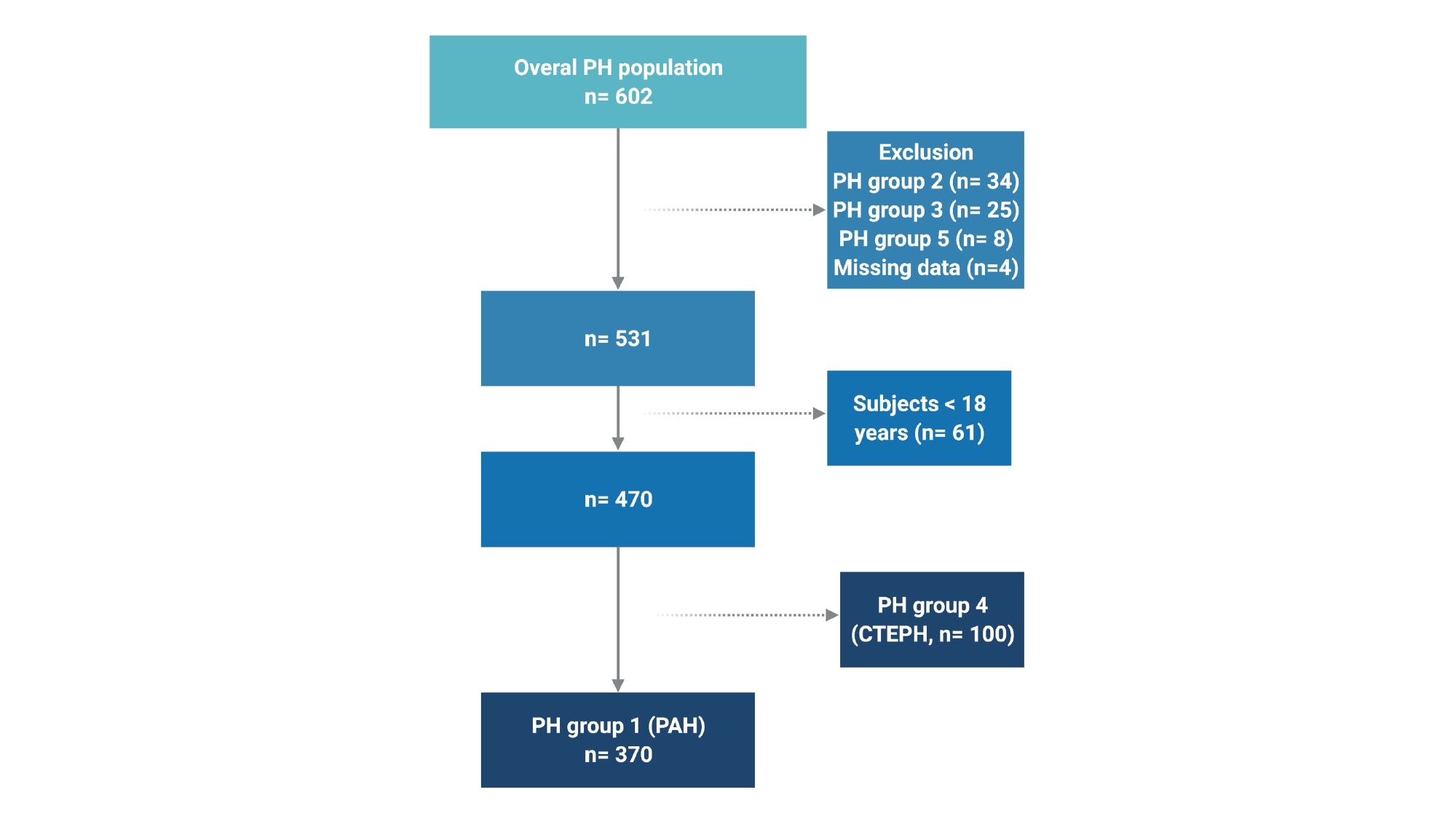

Supplement: Supplementary file 1 — Figurementary figure 1. [file PUL2-13-e12193-s001.jpg]
